# 

**Published:** 1890-03-01

**Authors:** 


### NEW OFFICES IN THE STRAND.

The Hospital *is now published at* **140, Strand, W.C.** *It has been found desirable to establish* The Hospital *in this central and convenient locality, owing to the rapid extension of the business of this paper of late.*

### NOTICE TO CORRESPONDENTS.

All MS., Letters, Books for Review, and other matters intended for the Editor should be addressed The Editor, The Lodge, Porchester Square, London, W.

**Notice to Advertisers and Subscribers.**

All Advertisements, Orders for Copies of The Hospital, and Business Communications should be addressed to The Publisher, *not to the Editor*, The Hospital, 140, Strand, W.C.

**Bound Volumes of "The Hospital."**

Vol. VI., containing full page portraits of T.R.H. the Prince and Princess of Wales, and Miss Florence Nightingale, is now ready, 4s. post free. Cases for binding may be had of the Publisher, at 1s. 6d. each.

**To Residents, Secretaries, and Matrons.**

The Editor will be glad to have the Regulations and each Report as issued by Asylums, Hospitals, Convalescent and Nursing Institutions, as well as those of other Charities, and an early copy in duplicate of all Appeals and Festival Notices, etc., addressed to *The Editor, The Lodge, Porchester Square, London, W.* Any superintendent, secretary, matron, or other chief official who regularly sends such information may be registered, on application to the Publisher, to receive free of cost a cover for binding The Hospital in half-yearly volumes.

### SCRAPS AND GLEANINGS.

SMALLPOX has broken out at Madeira.

NEW YORK has started a Pasteur Institute.

SPECIAL hospitals for consumption are to be built in Berlin.

THE Queen has sent a donation of £20 to the Cowes Compassionate fund.

THE influenza is re-appearing in Berlin; let us hope it will not re-appear in London.

LAUNCESTON ROWE DISPENSARY AND INFIRMARY treated 26 in-patients and 354 out-patients last year.

KINGSTON PROVIDENT DISPENSARY has over 3,000 members on its books, and reports continued progress.

HAMPSTEAD HOME HOSPITAL will hold a festival dinner at the Hotel Metropole on March 20th, the Lord Mayor in the chair.

DOGS are to be registered in Manchester, this seems a good scheme for the prevention of hydrophobia when muzzling has gone out.

LORD PORTMAN presided at the first meeting of the Blandford Cottage Hospital, and was able to move the adoption of a very satisfactory report.

THE quarterly court of governors of Middlesex Hospital met on Thursday. It is rumoured that Middlesex will have four dukes at its dinner this year.

THE late Mr. Samuel Fielden has bequeathed £3,000 to Manchester Royal Hospital, and £2,000 each to Brompton Hospital and the Royal Orthopædic Hospital.

THE authorities of St. Paul's Cathedral have been obliged to disperse the choir school for a few days in consequence of the prevailing epidemic and an attack of measles.

THE International Red Cross Committee of Geneva is collecting money for an "Augusta Fund," in recognition of the moral and material help given by the late Empress to the society.

DARLINGTON HOSPITAL treated 160 in-patients last year. A vote of thanks to the Misses Pease has been passed for their generosity in giving £550 for the structural additions to the hospital.

THERE is a strong feeling in the East-end against the cheap Swedish matches. Perhaps this is because Messrs. Bryant and May have their factories there and employ English workers only.

BLACKBURN AND EAST LANCASHIRE INFIRMARY has to raise £4,500 annually by voluntary subscriptions. The mill hands come forward nobly, and subscribe about £2,500, so that the infirmary is free from debt.

THE annual report of the London Fever Hospital says that 538 cases were under treatment during the year—males 229, females 309; 463 were discharged, 9 died, and 66 remained under treatment at the end of the year.

THE Burton-on-the-Water Cottage Hospital has treated less in-patients during 1889 than the average for the last 29 years, the number being 36; 101 out-patients were attended to. This hospital again has to report a favourable balance.

THE managers of the Leith General Hospital have granted the committee of the Surgeon Square Medical School for Women the exclusive use of the wards of the hospital for clinical study for their students for a period of three years.

THE report of the Committee of Management for 1889 of the Ledbury Cottage Hospital shows that out of 27 patients treated during the year, 26 were discharged cured. On receipts amounting to £204 18s. 9d., there is a balance in hand of £15 13s. 9d.

MRS. MARGARET ARNOT, of Kinnieswood Cottage, Earlston, who died on the 10th, has bequeathed £2 400 to various charities in Edinburgh—£500 to the Royal Infirmary, £300 to the Blind Asylum, and £300 to the Institution for the Deaf and Dumb.

NEW BRIGHTON has been rich in hospital meetings lately, the Convalescent Home, the Wallasey Cottage Hospital, and the Liscard District Association all report good progress. The Rev. C. Hylton Steward has been elected president of the Convalescent Home.

THE Directors of the Chalmers Hospital, Edinburgh, report that 295 in-patients were treated during the year, and 3,428 out-patients. A decided effort has been made to pay off the £265 4s. 6d. deficiency of last year, but there still remains a balance against the funds of £19 3s. 11d.

